# Efficient Whole-Cell Biocatalytic Transformation of Lignin-Derived Syringaldehyde to Syringic Acid with Aryl-Alcohol Oxidase in Deep Eutectic Solvent System

**DOI:** 10.3390/foods15020267

**Published:** 2026-01-12

**Authors:** Qing Li, Feng Li, Qi Wang, Ruicheng Yang, Zhe Zhang, Dian Dai, Zhangfeng Hu, Yucai He

**Affiliations:** 1 Institute of Microalgae Synthetic Biology and Green Manufacturing, School of Life Sciences, Jianghan University, Wuhan 430056, China; 24112090@jhun.edu.cn (Q.L.); fengli0702@jhun.edu.cn (F.L.); 242212101112@jhun.edu.cn (Q.W.); yangruicheng@jhun.edu.cn (R.Y.); zzhe@jhun.edu.cn (Z.Z.); 2 School of Pharmacy & School of Biological and Food Engineering, Changzhou University, Changzhou 213164, China; heyucai2001@163.com

**Keywords:** food preservatives, syringaldehyde, syringic acid, lignin, deep eutectic solvent

## Abstract

Syringic acid (SA) is a natural derivative of syringaldehyde (SD), derived from lignin depolymerization. Its application in the food industry focuses on the properties of natural functional ingredients; it is mainly used as a food antioxidant and food preservative, but can also be used as an ingredient to enhance food flavor and functional foods. This compound exhibits a remarkable spectrum of biological activities, including potent antioxidant, anti-inflammatory, neuroprotective, hypoglycemic, detoxifying, and anti-cancer effects, positioning it as a highly promising candidate for pharmaceutical and nutraceutical applications. In this study, suitable sites were first screened through homologous sequence alignment, and a variant of aryl-alcohol oxidase (CgAAO) with high efficiency in catalyzing the conversion of SD to SA was obtained via site-directed mutagenesis. A deep eutectic solvent (DES) system based on choline chloride/urea (ChCl/UR) in water was developed to enhance SA production. Additionally, key parameters of the biological reaction were optimized, including temperature, pH, metal ions, as well as the type and dosage of DES. The optimal performance was achieved using recombinant *E. coli* pRSFDuet-CgAAO-Y335F whole-cell biocatalysts, yielding 75% and producing 0.75 g/L SA in 100 mM KPB buffer (pH 7.0) containing 5 wt% ChCl/UR and 1 mM Fe^3+^. This study established a novel biosynthetic pathway for SA that was efficient, mild, green, and environmentally friendly.

## 1. Introduction

In recent years, the global environment faced increasingly severe challenges [1], frequent extreme weather events and the depletion of fossil fuels directed scientific attention [2], sustainable biological resources green biomanufacturing utilized renewable biomass to progressively replace fossil fuels and address the depletion of energy resources [3], thereby mitigating global ecological damage and supporting the steady development of a shared planetary future [4]. This study aimed to develop novel pathways for biomass utilization [5], converting biomass into high-value chemical products and offering new strategies for green biomanufacturing. Lignin, widely present in various plants, represented the most abundant and accessible organic carbon source on Earth [6]. It served as a promising alternative energy source, substantially reducing human reliance on non-renewable resources such as petroleum and coal [7].

Syringaldehyde, 4-hydroxy-3,5-dimethoxybenzaldehyde, occurs naturally as a lignin degradation product and is commonly found in clove, spruce, and bamboo leaves [8]. It also contributes a distinctive flavor to aged wines. SD functioned as a key intermediate in antibacterial agents such as trimethoprim in Bactrim and Biseptol [9]. Its synthesis primarily involves extraction from natural sources or chemical synthesis. When lignin undergoes minimal chemical transformation, it becomes more suitable for phenolic aldehyde production [10]. Tarabanko et al. examined the influence of lignin origin, process conditions, and pretreatment types on the formation of vanillin and SD during lignin oxidation. They identified a key competition between the condensation of syringyl and guaiacyl lignin fragments and their oxidative conversion to aldehydes [11]. Notably, Villar and coworkers reported a maximum combined yield of SD and vanillin of merely 14% from the nitrobenzene oxidation of lignin isolated from kraft black liquor via precipitation with calcium salts in a water-soluble alcohol. In contrast, nitrobenzene oxidation of lignin derived from rice straw proved more selective for these aldehydes, yielding SD and vanillin in a 1:1 ratio that represented a significant portion (50–59.7%) of the total detectable phenolic aldehydes [12].

In recent years, researchers have sought more efficient green organic solvents for lignin hydrolysis [13]. Deep eutectic solvents (DESs) emerged as a new class of organic solvent characterized by water miscibility, low volatility, non-flammability, biocompatibility, biodegradability, recyclability, low cost, and ease of preparation [14]. Compared to ionic liquids, DESs are less expensive and less toxic [15]. DESs entered the domains of chemical catalysis and biocatalysis [16]. In addition to their role as catalysts for the dehydration of fructose to 5-HMF, DESs also facilitate the subsequent derivatization and bioconversion of the resulting HMF [17]. Typically synthesized by heating a hydrogen bond acceptor (HBA) and donor (HBD) at a specific molar ratio (50–80 °C), acidic DESs have proven effective for HMF production [18]. Reported yields include 62% (Peng et al., 120 °C, 2 min), 68% (Wang et al., 170 °C), and 57% (Korner et al., 300 min) [19]. This versatility extended to biocatalysis in DES–aqueous systems. For instance, Peng et al. achieved near-quantitative (99.6%) bioconversion of HMF to BHMF using *E. coli* DCF over 48 h [20]. Wang et al. reported a 93.2% yield of HMFCA from HMF using whole-cell *E. coli* CV in a 3c-DES-H_2_O system at 30 °C within 12 h [21], a result mirrored by Zhang et al., who obtained a 98.4% yield of BHMF using *Pseudomonas putida S12* in a lactic acid:betaine:water DES under similar conditions [22].

Syringic acid, a naturally occurring phenolic compound, demonstrates considerable potential as a food additive, serving both as a preservative and an antioxidant. It offers a promising natural alternative to synthetic antioxidants like butylated hydroxyanisole (BHA) and butylated hydroxytoluene (BHT) [23]. Its primary mechanism involves inhibiting lipid oxidation and scavenging free radicals, thereby effectively extending the shelf life of fat-rich foods, meat products, and baked goods [24]. Furthermore, SA exhibits broad-spectrum antimicrobial activity against common foodborne pathogens, including *Escherichia coli* and *Staphylococcus aureus*, as well as spoilage molds such as *Penicillium* and *Aspergillus* [25]. This antimicrobial property supports its application in preserving a wide range of perishable foods, including fruits, vegetables, meat, and dairy products. Beyond its direct preservative functions, emerging research suggests that SA possesses several bioactive properties, such as anti-inflammatory and antibacterial effects, along with the potential to modulate gut microbiota. These attributes support its incorporation as a functional ingredient into health foods, probiotic formulations, and functional beverages, thereby enhancing the nutritional and health-promoting value of food products. Syringic acid, a natural phenolic organic compound and a benzoic acid derivative, exhibited high solubility in organic solvents but was insoluble in water [26]. These physicochemical properties complicated its production and isolation [27]. Recent studies revealed its significant potential in biomedical and health applications, including antioxidant, anti-inflammatory, neuroprotective, hypoglycemic, detoxifying, and anticancer activities [28,29,30]. Additionally, syringic acid acted as a natural food preservative. In cosmetics, it inhibited tyrosinase activity, reduced melanin accumulation, and provided a skin-lightening effect [31]. Although SA was widely distributed in plants, its low abundance and difficult extraction hindered large-scale industrial production. Chemical synthesis mainly involved methylation of gallic acid, but this reaction frequently generated byproducts such as incompletely methylated trimethoxybenzoic acid [32]. The biosynthetic route, oxidizing SD to SA, offered advantages such as mild reaction conditions and fewer side products.

**Figure 1:**
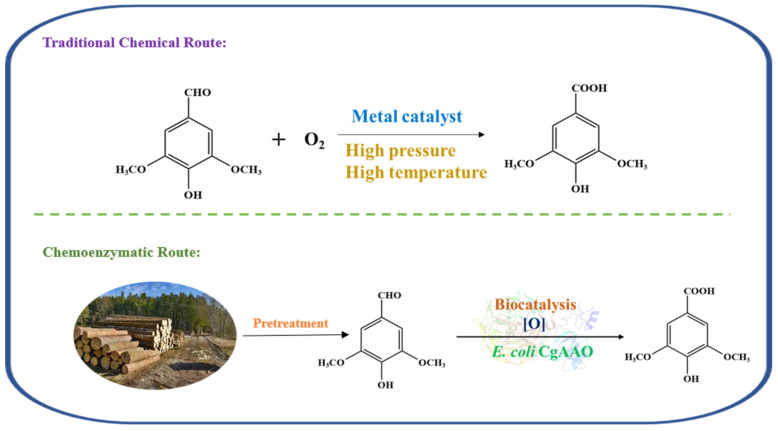
SD was oxidized to obtain SA through traditional chemical processes and chemical biological methods.

Chemical catalytic oxidation of SD employed Pt-C, Co/Mn/Br or MnOx-CeO_2_ as metal catalysts [33,34]. However, these conventional methods often required high temperature and pressure and suffered from product instability. Recently, biocatalysts such as unspecific peroxygenase (UPO), 5-hydroxymethylfurfural oxidase (HMFO) and fungal aryl alcohol oxidase (AAO) were applied to oxidize aromatic aldehydes under mild conditions [35]. An aryl alcohol oxidase from *C. graminicola* (CgAAO) attracts broad interest due to its high efficiency in catalyzing aryl aldehydes [33]. Through semi-rational design and machine learning, the thermal stability and solvent tolerance of wild-type CgAAO are enhanced [36,37,38]. Studies on its molecular dynamics and catalytic mechanism enable biotechnological routes for chemical synthesis, offering a green, economical, and environmentally promising strategy (Figure 1) [39,40].

In this study, target mutation sites were screened through homologous sequence alignment, and a CgAAO mutant capable of efficiently converting SD to SA was obtained by site-directed mutagenesis [41]. A DES ChCl/UR-water system was established, which increased the yield of syringic acid. Reaction conditions such as temperature, pH, metal ions, DES type, and dosage were optimized. Finally, a novel biosynthetic route for syringic acid was developed, characterized by high efficiency, mild conditions, and environmental friendliness. This work renders a new means of SA production from lignin-based chemicals, which has potential applications in the food, pharmaceutical and healthcare industries.

## 2. Materials and Methods

### 2.1. Materials

All strains (*E. coli* DH5α and BL21), plasmids, and primers (see Supplementary Files) were sourced from Shenggong in Wuhan, China (Table S1), while restriction enzymes and DNA polymerase were procured from Takara in Dalian, China. Chemicals and reagents (standard SD, standard SA, choline chloride, ethylene glycol, urea, glycerol, malonic acid, malic acid, ZnSO_4_, MgSO_4_, CoCl_2_, MnSO_4_, FeCl_3_, FeCl_2_, etc.), unless otherwise stated, were obtained from Sigma-Aldrich (St. Louis, MO, USA) or local suppliers at analytical grade. Cultivation media components, including LB broth and agar, were procured from Oxoid (Hampshire, UK). The water used in all experiments was deionized using a Milli-Q purification system.

### 2.2. Generation and Engineering of CgAAO Mutants in Recombinant E. coli

**Figure 2:**
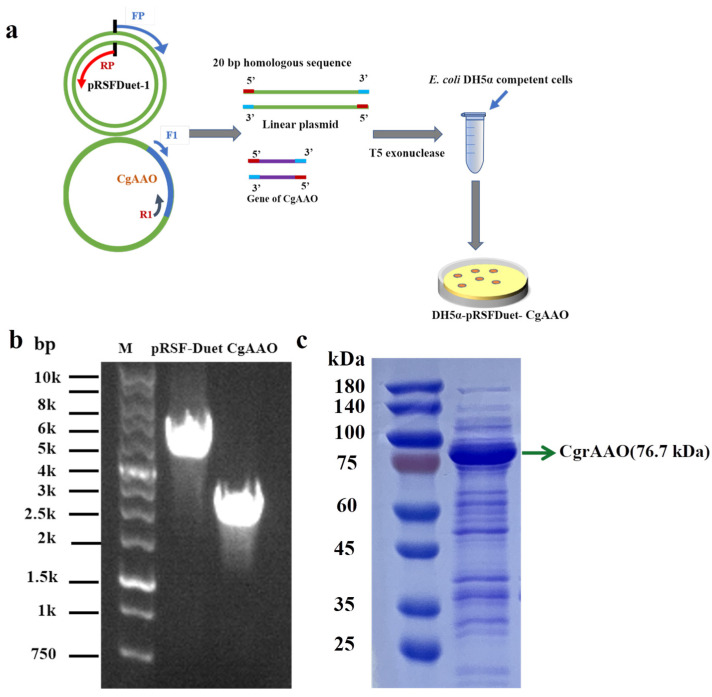
Key steps in the generation and verification of recombinant *E. coli* pRSFDuet-CgAAO, schematic of the construction method (**a**). Colony PCR verification of the CgAAO gene in *E. coli* DH5α transformants (M: DNA marker) (**b**). Confirmation of CgAAO protein expression in *E. coli* BL21 (DE3) by SDS-PAGE. Cells were normalized to OD_600_ = 10 before loading. Lane M: protein marker; Lane 1: soluble protein fraction (**c**).

The wild-type CgAAO was derived from *Colletotrichom graminicola* (Gene Bank number: EFQ27661.1, PDB number: 6RYV) [33], which had been reported to have certain catalytic activity towards aryl alcohols. However, after analysis, it was found that there is a signal peptide at the N-terminus of the enzyme, which was far from the active pocket and not conducive to *E. coli* recombinant overexpression. Therefore, a 21 amino acid segment could be cut off, and the signal peptide free CgAAO could be connected to the first cloning site of pRSFDuet using T5 nuclease ligation method, thus obtaining highly expressed pRSFDuet-CgAAO (Figure 2a). The mutation sites obtained through homologous comparison screening were analyzed using the error-prone PCR principle. A pair of primers containing the mutated amino acid was used to PCR the entire pRSFDuet-CgAAO to obtain the double stranded DNA of the mutant. Then, the Dpn I background wild-type plasmid was used to chemically transform the PCR linear double-stranded DNA into the *E. coli* DH5α clone strain for amplification and repair of DNA.

Recombinant *E. coli* cells were first inoculated into 4.0 mL of LB medium (10 g/L tryptone, 5 g/L yeast extract, 10 g/L NaCl) with 50 μg/mL kanamycin and grown at 37 °C for 3–4 h. This pre-culture (2 mL) was then used to inoculate 200 mL of TB medium (24 g/L yeast extract, 4 mL/L glycerol, 17 mM KH_2_PO_4_, 72 mM K_2_HPO_4_) containing 50 μg/mL kanamycin. Cells were grown at 37 °C and 220 rpm to an OD_600_ of 0.6–0.8. Protein expression was then induced with 0.50 mM IPTG, and the culture was incubated at 25 °C for 18 h. The induced culture was centrifuged (4000× *g*, 10 min, 4 °C) to harvest the cells. The cell pellet was washed twice with 100 mM potassium phosphate buffer (KPB, pH 8.0) and subsequently utilized as a whole-cell catalyst in biotransformation reactions.

### 2.3. Conversion and Optimization of SD to SA

This study systematically investigated the biocatalytic oxidation of SD to SA by *E. coli* CgAAO whole cells, encompassing key parameters such as temperature (15–40 °C), pH (5.5–8.0), metal ions (1 mM), DES species, DES concentration (0–60 vol%), and cell dose (0.1 g/L). Reactions were performed with 10 mM SD in 2 mL of medium. Aliquots were taken after incubation at 220 rpm, and SA yield and selectivity were analyzed by HPLC (Shimadzu LC-2030C 3D, Shimadzu, Kyoto, Japan) using the equations below:

(1) $$\mathrm{SA}\;\mathrm{yield}\;\left(\%\right)=\frac{\mathrm{SA}\;\mathrm{produced}\;(\mathrm{mM})}{\mathrm{Initial}\;\mathrm{SD}\;(\mathrm{mM})}\times 100$$

(2) $$\mathrm{SA}\;\mathrm{selectivity}\;(\%)=\frac{\mathrm{SA}\;\mathrm{produced}\;(\mathrm{mM})}{\mathrm{Initial}\;\mathrm{SD}\;(\mathrm{mM})}\times 100$$

### 2.4. Analysis Methods

SA and SD were analyzed using HPLC (Agilent Technologies 1200 series, Agilent, Santa Clara, CA, USA) with a Waters NovaPak C18 column (5 µm, 4.6 mm × 250 mm, Agilent, Santa Clara, CA, USA), column temperature of 40 °C, and mobile phase of methanol: 0.1% TFA (30:70) eluted at 0.8 mL/min. Both SA and SD have maximum absorption peaks at 280 nm (Figure S3). Figure S3 shows HPLC images of standards (SD and SA), SA liquor obtained from the biological oxidation of 5 mM SD in KPB (100 mM, pH 7.0), and SA liquor obtained from the biological oxidation of 5 mM SD in ChCl/UR–water (5:95, *v*/*v*). Note: The peak broadening for the authentic standards is attributed to their tautomeric properties under the applied analytical conditions. Peak identities were confirmed by spiking experiments.

## 3. Results

### 3.1. Assembly and Validation of a CgAAO Expression Strain in E. coli

To construct an *E. coli* strain expressing aryl alcohol oxidase (CgAAO) from *C. graminicola*, the corresponding gene was PCR-amplified and cloned into a linearized pRSFDuet-1 vector to yield the expression plasmid pRSFDuet-CgAAO (for primers, see Table S1). This plasmid was subsequently introduced into *E. coli* DH5α (Figure 2a), with successful expression of the target gene confirmed, as shown in Figure 2b. The amplification results confirmed the presence of the 2067 bp CgAAO insert and the recombinant plasmid. The production of the CgAAO protein, with an observed molecular weight of 76.7 kDa, was further validated by SDS-PAGE (Figure 2c).

### 3.2. Selection of Oxidase Catalysts and Screening of Mutation Sites

Oxidases were widely used in the preparation of high-value-added derivatives of acids; however, different sources of oxidases had different preferences for substrates. This study selected three different sources of oxidases based on the previous literature to explore the catalytic efficiency of oxidase types in generating syringic acid. The oxidases were sourced from *Mycobacterium sp*. MS 1601 (WP-083736035.1) (MycspAAO) [42], periplasmic aldehyde oxidase ABC (Gene ID:NDF79795.1) (PaoABC) [35], and aryl alcohol oxidase *C. graminicola* (CgAAO) (see Table S1). At a reaction temperature of 25 °C, in the reaction system with standard SD (5 mM) and KPB (100 mM) at pH 7.0, CgAAO without signal peptide exhibited the highest cellular biological oxidation activity. However, in comparison, CgAAO without signal peptide, MycspAAO, and PaoABC had only 30.8%, 68.5%, and 46.5% biological activity under the same conditions. Therefore, CgAAO without signal peptide can serve as the optimal biocatalyst for oxidative catalysis to generate syringic acid.

This study aims to construct a recombinant *E. coli* (CgAAO) strain derived from *C. graminicola* and perform site-directed mutagenesis to screen for highly active aromatic alcohol oxidase strains. Molecular docking of aromatic alcohol oxidase was used to predict whether mutation sites would affect the binding of substrates to active pockets. We used the analysis software PyMOL (version 1.5.0.5, www.pymol.org) to demonstrate the structure of CgAAO (PDB ID: 6RYV). Then, we used YASARA (http://www.yasara.org) by removing water molecules, surface charges, unbound ligand molecules, and metal ions.

**Figure 3:**
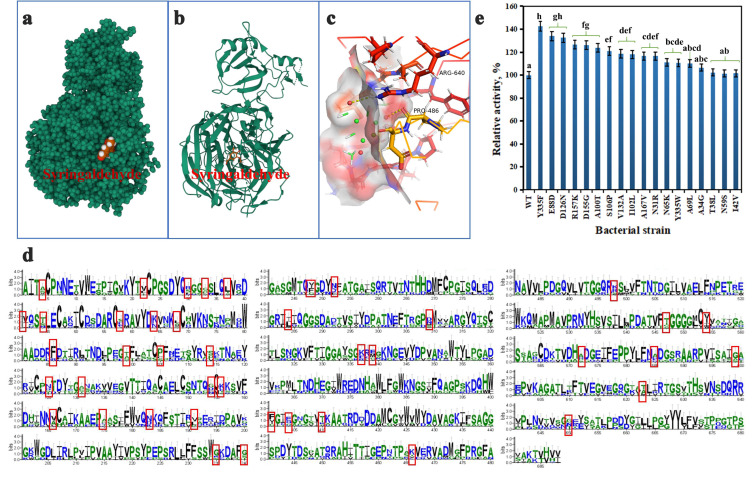
Computational insights into vanillin binding in CgAAO. Structural basis of vanillin docking within the three-dimensional framework of wild-type CgAAO (**a**). Molecular interactions of SD depicted in a ball-and-stick model (**b**). Key amino acid residues in the active pocket contributing to vanillin binding (**c**). Comparison of homologous sequences of 60 aromatic alcohol oxidase using WebLogo software. The total height of each stack in the figure represents the sequence conservation (information entropy) at that position, while the height of each individual letter corresponds to the occurrence frequency of its respective amino acid. Amino acids that differ between CgAAO and this consensus sequence are highlighted with red boxes. (**d**). Activity screening of mutants into 5 mM SD (**e**). Lowercase letters: significant difference comparison of the SA yield (*p* < 0.05).

The next step was to construct a docking lattice and optimize the energy of the ligand small molecule (vanillin) using YASARA. Finally, the monomer structure and ligand small molecules of aryl alcohol oxidase (CgAAO) were analyzed using YASARA software. The docking results (as shown in Figure 3a) indicate that the amino acids that interact with the furfural ligand within the 5 Å range are ARG640 and PRO486, respectively (Figure 3c). We used the consensus strategy to perform site-directed mutagenesis on wild-type CgAAO in order to construct more efficient and thermally stable mutant oxidase. It was generally observed from sequence statistics that more frequently occurring amino acids at specific positions tend to contribute more significantly to enzyme structural stability relative to their less frequent residues. Therefore, a consensus finder was used to find sequences similar to CgAAO. Firstly, we aligned the sequences to remove overexpressed sequences, then cropped the alignment of 60 homologous sequences to the size of the original query and generated a consensus. We calculated the count and frequency of each amino acid at each position corresponding to the input sequence. The positions where the input sequence differs from the consensus and the conservative threshold was higher than 60% are mutation sites. We obtained 18 suitable mutation sites, namely Y335, E88, D126, R157, D155, A100, S106, V132, I102, A167, N31, N65, Y335, A69, A34, T38, N59 and I42 (Figure 3d). In this study, a semi rational design was used to select mutation sites to improve the thermal stability and solvent tolerance of the aromatic alcohol oxidase CgAAO. A total of 18 sites were selected, resulting in 18 mutant strains. In a KPB 100 mM, pH 7.0 buffer solution, the mutant strain with the highest conversion efficiency was screened using 5 mM SD as the substrate. The wild-type could only produce 3.38 mM SA, with Y335F, E88D and D126N showing more significant advantages. The biological catalytic activity of the Y335F mutant strain was 1.43 times that of the wild-type (Figure 3e).

### 3.3. Optimization of Conditions for Biological Oxidation

**Figure 4:**
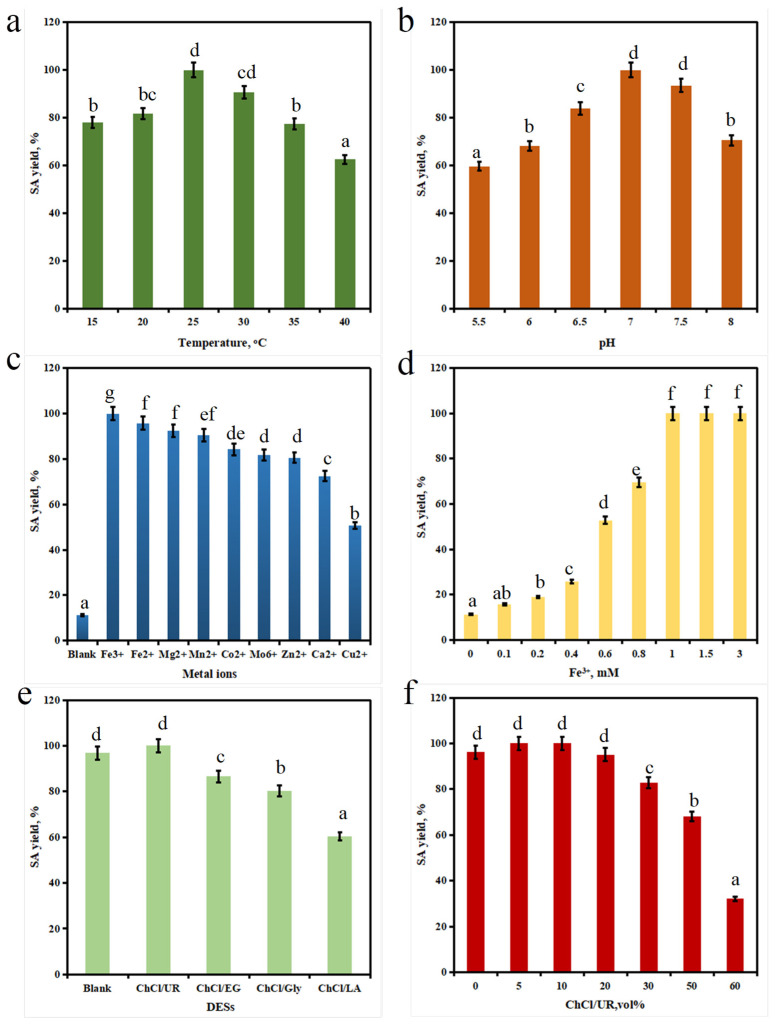
The SA yield of temperature (15–40 °C) on the biological oxidation of recombinant *E. coli* pRSFDuet-CgAAO-Y335F (KPB 100 mM, pH 7.0, reaction for 24 h). (**a**) The SA yield pH (5.5–8.0) on the biological oxidation of recombinant *E. coli* pRSFDuet-CgAAO-Y335F (25 °C, KPB 100 mM, pH 7.0, reaction for 24 h). (**b**) The SA yield of metal ion species (final concentration 1 mM) on the biological oxidation of recombinant *E. coli* pRSFDuet-CgAAO-Y335F (KPB 100 mM, pH 7.0, reaction time 24 h). (**c**) The SA yield of Fe^3+^ concentration (0–3 mM) on the biological oxidation of recombinant *E. coli* pRSFDuet-CgAAO-Y335F (KPB 100 mM, pH 7.0, reaction for 24 h). (**d**) The SA yield of DES type (added at 10% wt) on the biological oxidation of recombinant *E. coli* pRSFDuet-CgAAO-Y335F (1 mM Fe^3+^, KPB 100 mM, pH 7.0, reaction for 24 h). (**e**) The SA yield of DES ChCl/UR addition (0–60% wt) on the biological oxidation of recombinant *E. coli* pRSFDuet-CgAAO-Y335F (1 mM Fe^3+^, KPB 100 mM, pH 7.0, reaction time 24 h). (**f**) Lowercase letters: significant difference comparison of the SA yield (*p* < 0.05).

The temperature of biocatalytic reactions will affect the activity of enzymes, thereby affecting whole cell catalysis [43]. At pH 7.5, the activity of whole-cell catalytic oxidation reaction was detected using 10 mM SD as substrate at different temperatures (15–35 °C). The results showed that as the temperature gradually increased from 15 °C to 25 °C, the SA yield of whole-cell catalytic oxidation reaction increased from 77.9% to 100%. When the temperature of the reaction exceeded 25 °C, the SA yield of whole-cell catalytic oxidation reaction began to decrease (Figure 4a) because the activity of enzymes gradually froze and weakened under low-temperature conditions and increased with temperature. However, when the temperature was higher than the optimal reaction temperature, the three-dimensional structure of the enzyme, that was, the protein, would be destroyed, and the pocket of its active center would also change, resulting in a decrease in relative activity. However, when the temperature further increased, the structure of the enzyme would undergo irreversible damage, completely losing its activity [44]. Therefore, when the reaction temperature was 25 °C, the SA yield of whole-cell catalytic oxidation reaction reaches a maximum of 100%. As is well known, pH also had an impact on catalytic activity [45]. Our results showed that the reaction pH significantly affected the catalytic performance of the recombinant *E. coli* pRSFDuet-CgAAO whole cells. The SA yield was greatly enhanced as the pH increased from 5.5 to 7.0, with the maximum achieved at pH 7.0 (Figure 4b). Further increasing the pH to 8.0 caused a slight reduction in oxidative activity. This bell-shaped activity profile could be explained by the effect of pH on protein structure. Changes in pH could alter the charge distribution, leading to conformational rearrangements that affect the geometry of the catalytic active site and, consequently, reduce activity [46]. In conclusion, the optimum pH for the whole-cell catalytic oxidation was established as 7.0. In recent years’ research reports, metal ions played an indispensable role in biocatalytic reaction systems. Adding appropriate metal ions could effectively improve the activity of biocatalysts, which was likely related to the basic properties of enzymes themselves [47]. For example, Zn^2+^ and Co^2+^ could promote the SA yield of alcohol dehydrogenase in Sporidiobolus salicolor, but Co^2+^ and Cu^2+^ also had a certain inhibitory effect on LCR III short-chain dehydrogenase derived from the Lactobacillus curieae S1L19 strain [48]. This study investigated the effect of adding 1 mM of a series of common metal ions (Cu^2+^, Mg^2+^, Co^2+^, Fe^2+^, Zn^2+^, Mn^2+^, Fe^3+^, Mo^6+^, Ca^2+^ and Ni^2+^) to the oxidation reaction or in the city on the reaction activity. As shown in Figure 4c, it could be seen that Fe^2+^, Fe^3+^, Mg^2+^ and Mn^2+^ have a certain promoting effect on the oxidation reaction, while Co^2+^, Mo^6+^, Zn^2+^, Ca^2+^ and Cu^2+^ had a significant inhibitory effect on the SA yield of the oxidation reaction. Next, the effect of Fe^3+^ addition on the SA yield was also optimized, as shown in Figure 4c, because Fe^3+^ had a significant promoting effect on oxidation reaction and can be used as a metal ion additive in the reaction system. When the final concentration of Fe^3+^ in the system was 1 mM (Figure 4d), the SA yield reached its maximum, and there was no significant increase when it was further increased [33].

The solvents in the reaction system had a significant impact on biological oxidation, and the application of DESs as green solvents has also received increasing attention. DESs serve as environmentally friendly reaction media to enhance biocatalytic efficiency and are non-toxic and fully biodegradable [49]. They were a new type of green anhydrous solvent that had been successfully applied in the catalytic process of anhydrous enzymes after the generation of ionic liquids. These green solvents provided unique opportunities for studying protein overload, extending shelf life, enhancing enzyme activity, improving thermal stability, and sustainable bimolecular extraction processes [16]. Research by HsA et al. has shown that DESs can effectively enhance the catalytic activity and thermal stability of P450 BM-3. Zhang et al. used the foul-smelling pseudomonas S12 as a biocatalyst to convert 125 mM 5-hydroxymethylfurfural into 2,5-furandimethanol in a deep eutectic solvent water reaction medium, with a yield of up to 90.7% [22]. This study first investigated the effects of different types of DES and different dosages on the biological transamination reaction. Different ratios of DESs (ChCl/UR, ChCl/EG, ChCl/Gly, ChCl/LA) (5–60 wt%) and vanillin (5 mM) were added to a 1 mL reaction system. Under the optimized conditions (25 °C, pH 7.0, 1 mM Fe^3+^), the whole-cell biocatalytic performance of the recombinant aryl alcohol oxidase (CgAAO) mutant Y335F was evaluated. The results demonstrated that the addition of ChCl/UR significantly enhanced the yield of the oxidation reaction (Figure 4e). Specifically, a 5% (*v*/*v*) ChCl/UR supplementation led to a 1.2-fold increase in yield compared to the blank control (Figure 4f). In summary, the highest catalytic efficiency for the recombinant *E. coli* pRSFDuet-CgAAO whole-cell system was achieved in 100 mM KPB (pH 7.0) containing 1 mM Fe^3+^ and 5% (*v*/*v*) ChCl/UR.

### 3.4. Effect of SD Loading on Biological Oxidation

**Figure 5:**
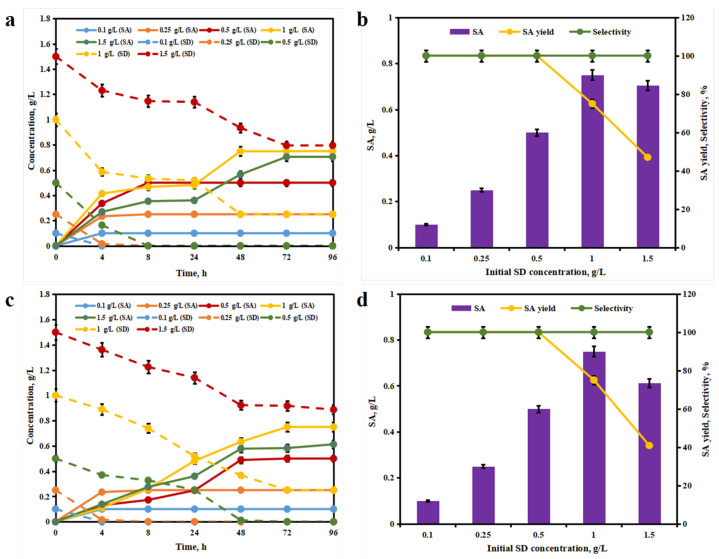
Time-course of SD and SA concentrations in the bioconversion system without deep eutectic solvent (DES). (**a**) SA concentration, yield, and selectivity as functions of initial SD concentration in the system without DES. (**b**) Time-course of SD and SA concentrations in the bioconversion system with choline chloride/urea (ChCl/UR) DES. (**c**) SA concentration, yield, and selectivity as functions of initial SD concentration in the system with ChCl/UR DES (**d**).

To investigate the effects of reaction systems and initial substrate concentration on SA production from SD, the time-course profiles of substrate and product concentrations, as well as SA yield and selectivity, were analyzed. Figure 5a,b correspond to the bioconversion process in pure 100 mM KPB buffer (pH 7.0). In Figure 5a, the time-course profiles show the dynamic changes in SD (dashed lines) and SA (solid lines) concentrations at different initial SD loadings. SD was rapidly consumed within the first 48 h, while SA accumulation increased steadily and reached a plateau thereafter. Figure 5b illustrated the dependence of SA concentration, yield, and selectivity on initial SD concentration. SA concentration and yield increased with increasing initial SD concentration, while selectivity remained consistently high (above 90%), indicating the efficient conversion of SD to SA in this system. Figure 5c,d represent the bioconversion process in a system supplemented with 5 wt% choline chloride/urea (ChCl/UR)-water DES. As shown in Figure 5c, similar to the pure KPB system, SD was degraded over time, and SA was produced accordingly, but the kinetic profiles and final concentrations differed, suggesting that the DES system modulated the bioconversion efficiency. Figure 5d shows that in the DES-containing system, SA concentration and yield also increased with initial SD concentration, while selectivity remained high. A comparison between the two systems (pure KPB vs. 5 wt% ChCl/UR-water) revealed that the DES addition altered the bioconversion kinetics and product accumulation, which would be further discussed in terms of reaction optimization and mechanism.

While this work demonstrates the high efficiency of the catalyst in converting SD to SA, it was important to note that the overall process yield from lignin remains unquantified. The reported metrics relate solely to the catalytic conversion step using a purified model compound. The full pathway yield from raw lignin would be heavily influenced by upstream processes—such as lignin depolymerization and SA separation—which were beyond the scope of this catalyst-focused study. Thus, future integration studies using lignin-derived streams are necessary to evaluate the complete process viability.

### 3.5. The Future Application of Biosynthetic Syringic Acid in Food Additives

**Figure 6:**
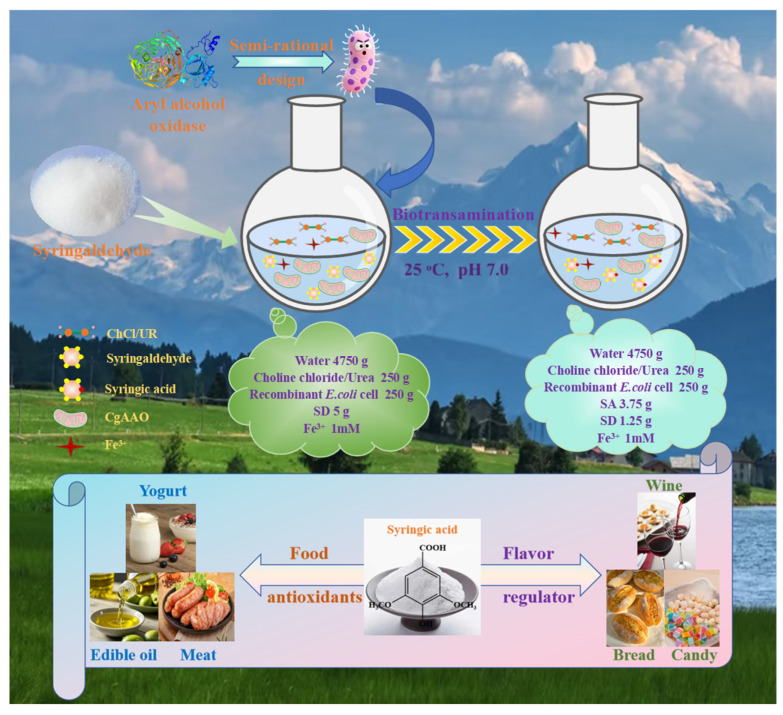
Mass balance from SD biological oxidation to SA in ChCl/UR-H_2_O.

The application of microbial biosynthesis for producing SA presents a sustainable and efficient strategy to secure this valuable phytochemical for the food industry. With demonstrated antioxidant and antimicrobial properties, biosynthesized SA serves as a multi-functional agent for food preservation and quality improvement. Its production via engineered *E. coli* offered a reliable and scalable supply, circumventing the drawbacks of conventional plant extraction. In application, SA’s potent free-radical scavenging activity makes it highly suitable for inhibiting lipid oxidation in edible oils, fats, and snack products, thereby preserving freshness and extending shelf life. In parallel, its antimicrobial efficacy against common foodborne bacteria and fungi supports its use as a natural preservative in products ranging from fresh produce to baked goods and meats. Integration methods could include direct food incorporation or functional packaging. The future of biosynthetic SA lies in leveraging synthetic biology to create tailored derivatives or production strains that optimize performance in food systems, aligning with clean-label trends. Critical steps toward commercialization include comprehensive safety assessment (e.g., GRAS designation), validation of efficacy in complex food matrices under practical conditions, and scaling studies to ensure cost-effectiveness. The convergence of biosynthesis and food technology through SA exemplified an innovative, green approach to addressing food spoilage, reducing waste, and enhancing product health benefits (Figure 6).

A critical consideration for the industrial translation of any biocatalytic process is the reusability and operational stability of the catalyst. While the present study successfully demonstrates the feasibility and efficiency of our whole-cell biocatalyst in the ChCl/UR system, the evaluation of catalyst reusability across multiple reaction cycles was beyond the scope of this initial proof-of-concept work. The high cell viability and sustained enzymatic activity observed in our single-batch experiments (Figure 3e) suggest the promising baseline stability of the biocatalyst in this non-conventional medium. Previous studies have reported the successful reuse of whole-cell systems in similar neoteric solvents, often facilitated by simple recovery methods such as centrifugation and washing. Future research must prioritize rigorous reusability tests, involving consecutive batch cycles with meticulous monitoring of activity retention. Furthermore, strategies to enhance robustness—such as cell immobilization on compatible solid supports or genetic engineering for improved solvent tolerance—could be explored to substantially extend the catalyst’s lifespan. Addressing this parameter is essential for performing a meaningful techno-economic analysis and demonstrating the true scalable potential of the proposed DES-based bioprocess.

In this context, the whole-cell catalyst design presented here offers inherent advantages for process improvement. By retaining the enzyme within a cellular matrix, we enhance operational stability, facilitate catalyst recovery and reuse, and reduce the need for extensive purification during the conversion stage—all factors that contribute to simplifying downstream processing. Looking forward, we agree that dedicated research into downstream purification strategies and comprehensive cost–benefit analysis is crucial. Our future work will specifically address the following: investigating compatible cell disruption or product separation techniques that maximize yield while maintaining process economics; evaluating the overall cost structure, including catalyst production, bioconversion efficiency, and downstream recovery, to identify key areas for optimization and transitioning from lab-scale validation to pilot-scale operations to assess performance under industrially relevant conditions.

### 3.6. Future Perspectives

While this study utilized commercial syringaldehyde to establish a fundamental understanding of the system, translating these findings into industrial applications necessitates addressing the inherent complexity of technical lignin feedstocks. Real lignins (e.g., Kraft lignin, organosolv lignin) contain inorganic salts, residual carbohydrates, and ash, which are expected to influence the efficacy of DES systems.

A primary challenge lies in feedstock impurities. Multivalent cations (e.g., Ca^2+^) may disrupt the hydrogen-bonding stability of the DES, thereby altering its solvation properties and viscosity. This could impede lignin dissolution and mass transfer. Concurrently, residual polysaccharides may undergo condensation reactions with target compounds, reducing the yield of SD. These factors collectively explain the typically lower yields and increased byproduct formation observed when using industrial lignin feedstocks compared to processes employing pure commercial SA.

To advance toward practical implementation, we propose two key strategies. First, feedstock pretreatment, such as intensive acid washing, could mitigate impurity interference. Second, developing more robust DES formulations—specifically tailored to tolerate specific impurity classes—would enhance system stability. Future work should focus on quantitatively assessing the lignin-depolymerization capability of the choline chloride/urea-ethylene glycol DES and evaluating its performance using pretreated lignin streams. This systematic approach is essential for critically evaluating the true scalability of DES-based lignin valorization processes.

## 4. Conclusions

In conclusion, this study successfully established a novel, efficient, and environmentally benign biosynthetic pathway for SA, a naturally derived compound with considerable therapeutic potential. By integrating protein engineering, which yielded a high-activity CgAAO-Y335F variant, with a ChCl/UR DES system, the research effectively enhanced the catalytic conversion from SD to SA. Key reaction parameters, including pH, temperature, metal ion concentration, and DES dosage, were systematically optimized. Ultimately, employing recombinant *E. coli* whole cells as biocatalysts under the optimized conditions, the system achieved a 75% yield and a concentration of 0.75 g SA per liter of reaction solution. SA possesses a definitively superior biological safety profile relative to many prevalent synthetic antioxidants used in food preservation. This conclusion was supported by its natural derivation from edible biomass, its established presence in the human diet, and toxicological data suggesting a wider safety margin. Unlike some synthetic options associated with potential health controversies, SA’s favorable safety parameters facilitate its potential approval for broader food applications. Therefore, its adoption not only addressed safety concerns but also supported the development of more sustainable and consumer-acceptable food preservation strategies, marking a significant step towards cleaner and safer food production systems. This integrated approach demonstrated a mild and sustainable production strategy, offering a viable green alternative to conventional synthesis methods and facilitating the future scalable production of SA from biomass-based chemicals for food, pharmaceutical and healthcare applications.

## Data Availability

The original contributions presented in the study are included in the article/Supplementary Materials. Further inquiries can be directed to the corresponding authors.

## Supplementary Materials

The following are available online at https://www.mdpi.com/article/10.3390/foods15020267/s1: Table S1: Strain, plasmids, and primer used in this study. Table S2: The nucleotide sequences of optimized—CgAAO encoding genes. Figure S1: Image of recombinant plamid. Figure S2: Biological oxidation of SD with different oxidases biocatalysts (CgAAO-WT, CgAAO, MyAAO, PaoABC) at 25 °C and pH 7.0. Figure S3: (a) HPLC chromatogram of the SD standard. (b) HPLC chromatogram of the SA standard. (c) SA liquor obtained from the biological oxidation of 5 mM SD in KPB (100 mM, pH 7.0). (d) SA and SD liquor obtained from the biological oxidation of 5 mM SD in ChCl/UR–water (5:95, *v*:*v*). Note: The peak broadening for the authentic standards is attributed to their tautomeric properties under the applied analytical conditions. Peak identities were confirmed by spiking experiments.

## Abbreviations

The following abbreviations are used in this manuscript:

SA	Syringic acid
SD	Syringaldehyde
DES	Deep Eutectic Solvent
ChCl/UR	Choline Chloride/Urea
CgAAO	Aryl alcohol oxidase from *C. graminicola*
UPO	Unspecific peroxygenase
HMFO	5-Hydroxymethylfurfural oxidase
AAO	Aryl alcohol oxidase
MycspAAO	Oxidases were sourced from *Mycobacterium* sp. MS 1601
PaoABC	Periplasmic aldehyde oxidase ABC
