# Supplementary material for: Efficient Whole-Cell Biocatalytic Transformation of Lignin-Derived Syringaldehyde to Syringic Acid with Aryl-Alcohol Oxidase in Deep Eutectic Solvent System
Source: Foods. 2026 Jan 12;15(2):267. doi: 10.3390/foods15020267 (PMC12839953; doi:10.3390/foods15020267)
Supplement: Supplementary file 1 [file foods-15-00267-s001.zip › foods-4049512-supplementary.pdf]

foods MDPI

Support Information

Table S1. Strain, plasmids, and primer used in this study.

Strain, plasmid or primer	Description or sequence (5′-3′)	Source
Strain		
Escherichia coli DH5α	gene cloning strain	Novagen (Shanghai, China)
Escherichia coli BL21 (DE3)	protein expression strain	Novagen (Shanghai, China)
Plasmid		
pRSFDuet-1	Kan^+	Novagen (Germany)
Primer		
pRSFDuet-F	AGTTTGAAAAATAAAAGCTTGCGGCCGCAT AATGCTTAAGTCGAAC	Shenggong (Wuhan, China)
pRSFDuet-R	GTGCGCTTGCCATGGGATCCTGGCTGTGGTG ATGATGGTGATGGC	
CgAAO-F	ATGGCAATTACCAGTTGCCCGAATAATGA	Shenggong (Wuhan, China)
CgAAO-R	GTATTGCAAAGACCGTTCATGTTATT	

Table S2. The nucleotide sequences of optimized CgAAO encoding genes.

Name	Sequences
CgAAO	ATGGCAATTACCAGTTGCCCGAATAATGAAACCGTGTGGGAAACCCCGATTGGCGTTAAATAT<br>ACCCTGTGCCCTGGTAGTGATTATCAGAATGGTGGCGCCAGTCTGCAGACCGTGCGCGATATT<br>CAGAGTAGTCTGGAATGTGCCAAAATTTGTGATAGTGATGCCCGTTGCAATCGCGCCGTGTAT<br>GATAATGTTAATAAGGCCTGCGATGTTAAGAATAGCACCAATCCGATGCAGTGGGCAGCAGA<br>TGATCGCTTTGAAACCATTCGTCTGACCAATGATCTGCCGGAAGGTGCCTTTATTAGCACCTGT<br>AGCTTTAATGAAACCAGCTATCGTGTGCCGGAAACCAATGCAGAATATCGCATTTGCCCGGAT<br>ACCGATTATACCGGTGTTAATGCAAAAGTTGTTGAAGGTGTGACCACCATTCAGGCCTGTGCC<br>GAACTGTGTAGTAATACCCAGGATTGCCGTAAAAGCGTGTTTGATCATATTAATAACGCCTGT<br>GCCATTAAGGCCGCAGAACCGGCAACCAGCATTTTCTGGGTTCAGGATAAACAGTTTAGTACC<br>ATTCGCCTGCCGGAAAATATTGATCCGGCCGTGAAAGGCAAATGGGGCGATCTGATTCGTCTG<br>CCGGTTATTCCGGTGGCCGCATATATTGTGCCGAGCTATCCGGAACCGAGTCGTCTGCTGTTCT<br>TTAGCAGTTGGAGCAATGATGCATTTTCAGGCGCAAGCGGTATGACCCAGTTTGGTGACTATG<br>ATTTTGCCACCGGCGCCATTAGTCAGCGTACCGTGACCAATACCCATCATGATATGTTTTGCCC<br>TGGTATTAGTCAGCTGGAAGATGGTCGCATTCTGATTCAGGGTGGCAGCGATGCCGATACCGT<br>TAGTATCTATGATCCGGCCACCAATGAGTTTACCCGTGGTCCGAATATGACCCTGGCACGTGGT<br>TATCAGACCAGCTGCACCCTGAGTAATGGTAAAGTGTTTACCATTGGTGGCGCATATAGCGGC<br>GAACGCGTTGGCAAGAATGGTGAAGTGTATGATCCGGTTGCAAATGCATGGACCTATCTGCCG<br>GGCGCCGATTTTCGCCCGATGCTGACCAATGACCATGAAGGCATTTGGCGTGAAGATAATCAT<br>GCCTGGCTGTTTGGTTGGAAGAATGGTAGCATTTTCCAGGCAGGCCCGAGCAAAGATCAGCAT<br>TGGTATGGCATTCAGGGCAATGGCACCGTTGCAAAAGCCGCAACCCGTGATGATGATGATGC<br>AATGTGTGGCGTTTGGGTTATGTATGATGCCGTTGCAGGTAAAATTTTCAGCGCAGGTGGCAGT<br>CCGGATTATACCGATAGCCCGGCAACCCAGCGCGCCCATATTACCACCATTGGTGAACCGAAT<br>ACCCCGGCAGAAGTGGAACGTGTTGCCGATATGGGCTTTCCGCGTGGTTTTGCAAATGCAGTG<br>GTGCTGCCGGATGGTCAGGTTCTGGTGACCGGCGGTCAGCGTATGAGCCTGGTGTTTACCAAT<br>ACCGATGGTATTCTGGTGGCAGAACTGTTTAATCCGGAAACCCGCGAATGGAAACAGATGGC<br>CCCGATGGCAGTGCCGCGTAATTATCATAGTGTTAGCATTCTGCTGCCGGATGCAACCGTGTTT<br>AGCGGCGGCGGTGGCATGTGTTGGGTTCAGAATGTTGGTGACAGCACCGCCGGTTGTGATAAA<br>ACCGTTGATCATAGTGATGGTGAAATTTTCGAACCGCCGTATCTGTTTAATGAAGATGGTAGCC<br>GTGCCGCCCGTCCGGTTATTAGCGCCATTAGTGCCGATCCGATTAAGGCAGGTGCCACCCTGA<br>CCTTTACCGTGGAAGGCGTGGAAGGTCAGGGCACCGCCGCCTTAATTCGTCTGGGCAGTGTGA<br>CCCATAGTGTTAATAGCGATCAGCGTCGTGTGCCGCTGAATGTGACCGTGAGTGGCAATGAAT<br>ATAGCGCAACCCTGCCGGATGATTATGGTATTCTGCTGCCTGGTTATTATTATCTGTTTGTTAGC<br>ACCCCGCAGGGTACCCCGAGTATTGCAAAGACCGTTCATGTTATT

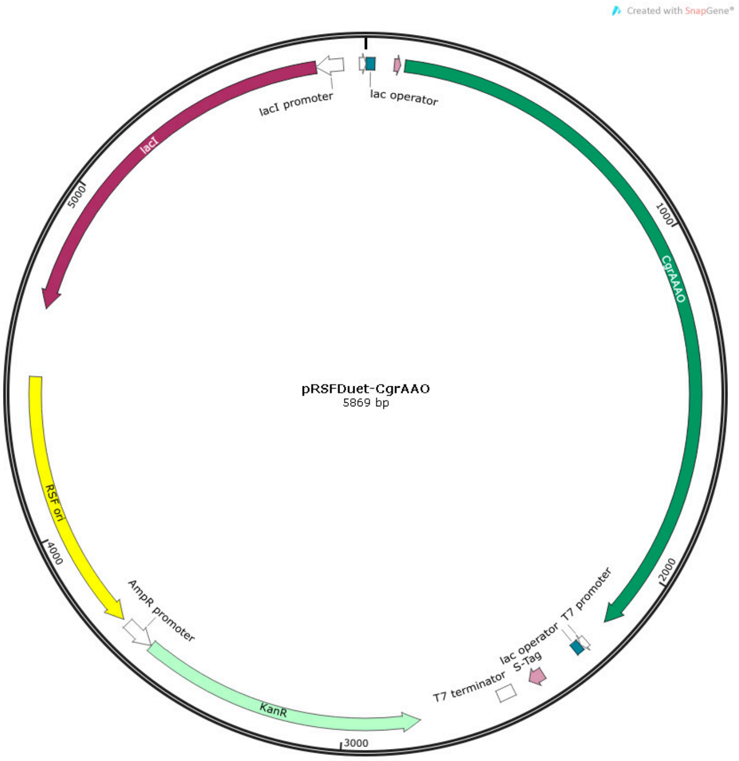

Figure S1. Image of recombinant plamid

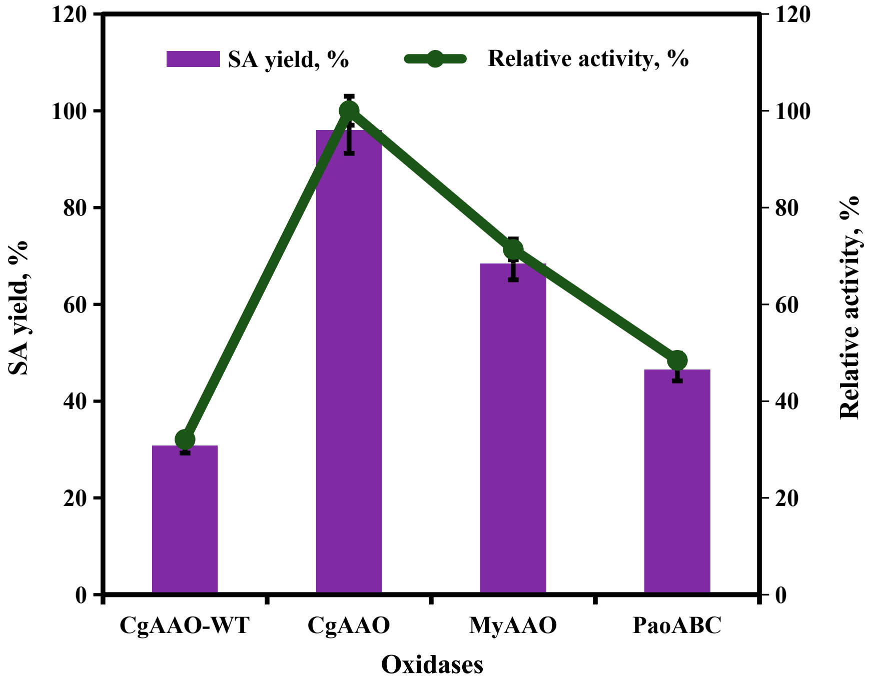

Figure S2. Biological oxidation of SD with different oxidases biocatalysts (CgAAO-WT, CgAAO, MyAAO, PaoABC) at 25 °C and pH 7.0

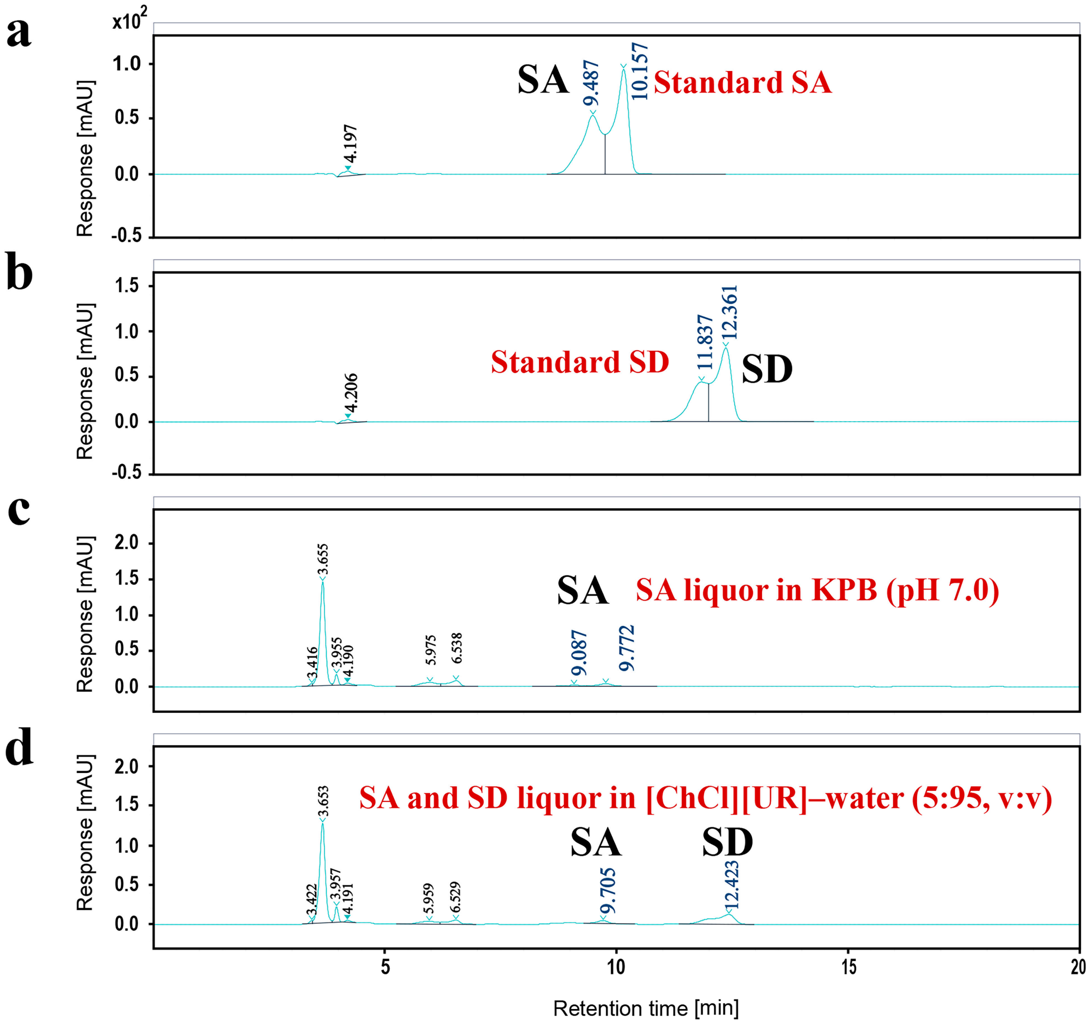

Figure S3. (a) HPLC chromatogram of the SD standard. (b) HPLC chromatogram of the SA standard. (c) SA liquor obtained from the biological oxidation of 5 mM SD in KPB (100 mM, pH 7.0). (d) SA and SD liquor obtained from the biological oxidation of 5 mM SD in ChClUR–water (5:95, *v:v*). Note: The peak broadening for the authentic standards is attributed to their tautomeric properties under the applied analytical conditions. Peak identities were confirmed by spiking experiments.
